# LogTODIM-PROMETHEE technique for development evaluation of school-enterprise cooperation from the perspective of collaborative education based on the probabilistic linguistic group decision-making

**DOI:** 10.1016/j.heliyon.2024.e33391

**Published:** 2024-06-21

**Authors:** Yuedan Hu, Chalermchai Panyadee

**Affiliations:** School of Administrative Studies, Maejo University, 50290, Chiangmai, Thailand

**Keywords:** Multiple-attribute group decision-making (MAGDM), Probabilistic linguistic term sets (PLTSs), Logarithmic TODIM technique, PROMETHEE technique, Development evaluation of school-enterprise cooperation

## Abstract

The integration of industry and education can be literally explained as the role of education in providing intellectual support and production services for the development of industries in a country or region, evolving from an auxiliary role in industrial development to a supporting role in industrial development. The government, enterprises, universities, and intermediaries are all the main bodies of industry education integration, and school enterprise cooperation is the core. The two basic functions of universities, namely “applied scientific research” and “social services”, must be highlighted. How to strengthen the cultivation of applied talents and improve the quality of talent cultivation in universities is a problem that universities must face in their transformation. Strengthening school enterprise cooperation and promoting the integration of industry and education is an effective way to solve this problem. The development evaluation of school-enterprise cooperation from the perspective of collaborative education is a multiple-attribute group decision-making (MAGDM) problem. Recently, the Logarithmic TODIM (LogTODIM) and PROMETHEE technique was employed to put forward the MAGDM issues. The probabilistic linguistic term sets (PLTSs) are employed as a technique for characterizing uncertain information during the development evaluation of school-enterprise cooperation from the perspective of collaborative education. In this paper, the probabilistic linguistic LogTODIM-PROMETHEE (PL-LogTODIM-PROMETHEE) technique is constructed to put forward the MAGDM under PLTSs. The MEREC technique is employed to obtain the weight values under PLTSs. Finally, a numerical example for development evaluation of school-enterprise cooperation from the perspective of collaborative education is put forward to validate the LogTODIM-PROMETHEE technique.

## 1 Introduction

The current mode of school enterprise cooperation in our country is quite diverse, which is a characteristic path that is constantly evolving according to our national conditions and actual development needs. School enterprise cooperation is an important educational approach that has evolved from traditional education to education oriented towards society, market, and enterprises, cultivating professional and technical talents [[1], [2], [3], [4], [5]]. However, it has not yet fully established a win-win path for the government, schools, enterprises, and industries. The degree of participation of enterprises in school enterprise cooperation in developed countries abroad is very high, while in China, most of the cooperation is still superficial [[6], [7], [8], [9], [10]]. The system of school enterprise cooperation is not perfect enough, the level of “dual teacher” talent construction still needs to be improved, and the concept of school enterprise cooperation in China has not been completely changed. These all bring difficulties to the further deep integration of schools and enterprises. In order to implement precise policies and effectively solve problems, the first step is to further improve the mechanism of school enterprise cooperation in the system, which can refer to Finland's system of signing performance agreements between applied technology universities and the Ministry of Education and Culture [[11], [12], [13], [14], [15]]. The general basis for central government funding to universities is based on the number of students on campus, but if the reform is based on financial allocations such as teaching completion rate, student training quality, and research quality, it will promote the deepening reform of school enterprise cooperation [[16], [17], [18], [19]]. Play the intermediary role of industry associations, which are non-profit organizations composed of independent business units that protect and promote the established interests of all members. Industry associations are familiar with the needs of both schools and enterprises, and can serve as a bridge for information communication in school enterprise cooperation. Led by industry associations, a specialized cooperation management mechanism can be established to develop characteristic cooperation plans based on the actual situation of schools and enterprises, which can provide targeted solutions and promote school enterprise cooperation more smoothly [[20], [21], [22], [23]]. The concept of school enterprise cooperation needs to be appropriately transformed. In the era of intelligence, school enterprise cooperation should pay attention to human centered demands in educating people [[24], [25], [26], [27]]. If it is only to improve employment rates, it will ultimately make the trained people “out of the race against machines.”. We should have a broad vision, strengthen the cultivation of key abilities and ways of thinking, focus on exploring the connection between future technological development direction and deep integration of industry and education, and strive to cultivate high-quality composite talents that meet the needs of future socio-economic development. In this regard, we can take the innovation and entrepreneurship competition as an opportunity to address the current situation of shallow, scattered, and unsystematic innovation and entrepreneurship education [[28], [29], [30]]. We can make good use of school enterprise cooperation platforms to guide the design of innovation and entrepreneurship courses and build a curriculum system that fosters innovation and entrepreneurship awareness; Provide dual qualified part-time teachers to alleviate the embarrassment of lacking innovative and entrepreneurial teachers in universities; Jointly building a school enterprise innovation and entrepreneurship practice platform, allowing students to accumulate innovation and entrepreneurship experience in a simulated entrepreneurial environment, and fully integrating the spirit of entrepreneurship and entrepreneurship into classroom teaching, practical education, and project cooperation, enhancing students' awareness of innovation and entrepreneurship [[31], [32], [33], [34]]. Relying on the new opportunities of promoting the construction of industrial colleges and future technical colleges by the country, we continuously expand the new connotations of school enterprise cooperation, cultivate talents with more industry characteristics, and jointly enhance the social influence of both schools and enterprises [[35], [36], [37], [38], [39]].

With the increasing complexity of economic and social issues, as well as the inherent thinking ambiguity and cognitive limitations of decision-makers, it is becoming increasingly difficult for them to express their evaluation information of objective things with precise numerical values [[40], [41], [42], [43], [44]]. To effectively describe the uncertainty evaluation information of decision-makers, Professor Zadeh [45] proposed the concept of fuzzy sets (FSs) in 1965, which provides a good approach and method for dealing with and solving complex uncertainty decision problems. As is well known, decision-making is ubiquitous in life [[46], [47], [48], [49]]. In the past, people often made decisions based on knowledge, experience, and other aspects when making decisions or when we usually made decisions on things with relatively small interests [[50], [51], [52]]. However, nowadays, the factors we need to consider when making decisions are becoming increasingly numerous and difficult to estimate in terms of importance [[53], [54], [55], [56]]. The relationships between attributes are also becoming increasingly close, especially at the level of enterprises and countries. MADM is becoming increasingly important [[57], [58], [59], [60]]. At this point, if only experience is used for decision-making, the enterprise or country will face enormous risks [61]. How to combine scientific theoretical methods to make decisions is an important research question. The development evaluation of school-enterprise cooperation from the perspective of collaborative education is a MAGDM problem. Recently, the LogTODIM [62,63] and PROMETHEE technique [[64], [65], [66]] has been employed to put forward the MAGDM issues. The probabilistic linguistic term sets (PLTSs) [67] are employed as a technique for characterizing uncertain information during the development evaluation of school-enterprise cooperation from the perspective of collaborative education. Furthermore, many techniques employed the LogTODIM [62,63] and PROMETHEE technique [64] to put up with the MAGDM problem. Until now, no or few techniques have been constructed on MEREC technique [68] and LogTODIM-PROMETHEE technique under PLTSs. Therefore, in this paper, the PL-LogTODIM-PROMETHEE technique is constructed to put forward the MAGDM under PLTSs. The MEREC technique is employed to obtain the weight values under PLTSs. Finally, a numerical example for development evaluation of school-enterprise cooperation from the perspective of collaborative education is put forward to validate the PL-LogTODIM-PROMETHEE technique.

The structure of this paper is put forward below. In Sect. 2, the PLTSs is put forward. In Sect. 3, PL-LogTODIM-PROMETHEE technique is put forward under PLTSs with entropy. Sect. 4 puts forward the numerical example for development evaluation of school-enterprise cooperation from the perspective of collaborative education and some comparative analysis. Some remarks are put forward in Sect. 5.

## 2 Preliminaries

Pang et al. [69] put forward the PLTSs.

Definition 1 [69]. If $GL=\left\{gl_{g\alpha }|g\alpha =-g\theta ,\cdots ,-2,-1,0,1,2,\cdots g\theta \right\}$ is the LTS, the $gl_{g\alpha }$ depict the information to π with function gh in Eq. (1):

(1) $$\begin{array}{l} gh:\left[gl_{-g\theta },gl_{g\theta }\right]\to \left[0,1\right]\text{,}\\ gh\left(gl_{g\alpha }\right)=\frac{g\alpha +g\theta }{2g\theta }=\pi \end{array}$$

π is denoted the $gl_{g\alpha }$ with function $gh^{-1}$ in Eq. (2):

(2) $$\begin{array}{l} gh^{-1}:\left[0,1\right]\to \left[gl_{-g\theta },gl_{g\theta }\right]\text{,}\\ gh^{-1}\left(\pi \right)=gl_{\left(2\pi -1\right)g\theta }=gl_{g\alpha } \end{array}$$

Definition 2 [69]. If $GL=\left\{gl_{g\alpha }|g\alpha =-g\theta ,\cdots ,-2,-1,0,1,2,\cdots g\theta \right\}$, the PLTS is constructed in Eq. (3):

(3) $$GL\left(gp\right)=\left\{gl^{\left(g\varphi \right)}\left(gp^{\left(g\varphi \right)}\right)|\left(\begin{array}{l} gl^{\left(g\varphi \right)}\in GL,gp^{\left(g\varphi \right)}\geq 0,\\ g\varphi =1,2,\cdots ,\#GL\left(gp\right),\sum_{g\varphi =1}^{\#GL\left(gp\right)}gp^{\left(g\varphi \right)}\leq 1 \end{array}\right)\right\}$$

where $gl^{\left(g\varphi \right)}\left(gp^{\left(g\varphi \right)}\right)$ is the $g\varphi \text{th}gl^{\left(g\varphi \right)}$ with probability values $gp^{\left(g\varphi \right)}$, and #GL(gp) is the length of #GL(gp). The $gl^{\left(g\varphi \right)}$ in GL(gp) are depicted by employing the ascending order.

Pang et al. [69] normalized the PLTS GL(gp) as

$$GL\left(gp\right)=\left\{gl^{\left(g\varphi \right)}\left(gp^{\left(g\varphi \right)}\right)|\left(\begin{array}{l} gl^{\left(g\varphi \right)}\in GL,gp^{\left(g\varphi \right)}\geq 0,\\ g\varphi =1,2,\cdots ,\#GL\left(gp\right),\sum_{g\varphi =1}^{\#GL\left(gp\right)}gp^{\left(g\varphi \right)}=1 \end{array}\right)\right\}$$

where $gp^{\left(g\varphi \right)}=gp^{\left(g\varphi \right)}/\sum_{g\varphi =1}^{\#GL\left(gp\right)}gp^{\left(g\varphi \right)}$ for all gφ=1,2,⋯,#GL(gp).

Definition 3 [69]. If $GL=\left\{gl_{g\alpha }|g\alpha =-g\theta ,\cdots ,-2,-1,0,1,2,\cdots g\theta \right\}$ is LTSs, $GL_{1}\left(gp\right)=\left\{g{l_{1}}^{\left(g\varphi \right)}\left(g{p_{1}}^{\left(g\varphi \right)}\right)|\;g\varphi =1,2,\cdots ,\#GL_{1}\left(gp\right)\right\}$ and $GL_{2}\left(gp\right)=\left\{g{l_{2}}^{\left(g\varphi \right)}\left(g{p_{2}}^{\left(g\varphi \right)}\right)|\;g\varphi =1,2,\cdots ,\#GL_{2}\left(gp\right)\right\}$ be two PLTSs, $\#GL_{2}\left(gp\right)$ and $\#GL_{2}\left(gp\right)$ are the length of $GL_{1}\left(gp\right)$ and $GL_{2}\left(gp\right)$. If $\#GL_{1}\left(gp\right)>\#GL_{2}\left(gp\right)$, then add $\#GL_{1}\left(gp\right)-\#GL_{2}\left(gp\right)$ LTSs to $GL_{2}\left(gp\right)$. The added LTSs are the smallest LTS in $GL_{2}\left(gp\right)$ and the probabilities are zero.

Definition 4 [69]. If $GL=\left\{gl_{g\alpha }|g\alpha =-g\theta ,\cdots ,-2,-1,0,1,2,\cdots g\theta \right\}$ is LTSs, For the $GL_{1}\left(gp\right)=\left\{g{l_{1}}^{\left(g\varphi \right)}\left(g{p_{1}}^{\left(g\varphi \right)}\right)|\;g\varphi =1,2,\cdots ,\#GL_{1}\left(gp\right)\right\}$ and $GL_{2}\left(gp\right)=\left\{g{l_{2}}^{\left(g\varphi \right)}\left(g{p_{2}}^{\left(g\varphi \right)}\right)|\;g\varphi =1,2,\cdots ,\#GL_{2}\left(gp\right)\right\}$, the expected values $EV\left(GL_{1}\left(gp\right)\right),EV\left(GL_{2}\left(gp\right)\right)$ and deviation degree $DD\left(GL_{1}\left(gp\right)\right),DD\left(GL_{2}\left(gp\right)\right)$ of $GL_{1}\left(gp\right)$ and $GL_{2}\left(gp\right)$ is constructed in Eq.(4–5):

(4-a) $$EV\left(GL_{1}\left(gp\right)\right)=\sum_{g\varphi =1}^{\#GL_{1}\left(gp\right)}gh\left(GL_{1}\left(gp\right)\right)gp^{\left(g\varphi \right)}/\sum_{g\varphi =1}^{\#GL_{1}\left(gp\right)}gp^{\left(g\varphi \right)}$$

(4-b) $$EV\left(GL_{2}\left(gp\right)\right)=\sum_{g\varphi =1}^{\#GL_{2}\left(gp\right)}gh\left(GL_{2}\left(gp\right)\right)gp^{\left(g\varphi \right)}/\sum_{g\varphi =1}^{\#GL_{2}\left(gp\right)}gp^{\left(g\varphi \right)}$$

(5-a) $$DD\left(GL_{1}\left(gp\right)\right)=\sqrt{\sum_{g\varphi =1}^{\#GL_{1}\left(gp\right)}{\left(gh\left(GL_{1}\left(gp\right)\right)gp^{\left(g\varphi \right)}-EV\left(GL_{1}\left(gp\right)\right)\right)}^{2}}$$

(5-b) $$DD\left(GL_{2}\left(gp\right)\right)=\sqrt{\sum_{g\varphi =1}^{\#GL_{2}\left(gp\right)}{\left(gh\left(GL_{2}\left(gp\right)\right)gp^{\left(g\varphi \right)}-EV\left(GL_{2}\left(gp\right)\right)\right)}^{2}}$$

The order for two PLTSs is constructed: (1) if $EV\left(GL_{1}\left(gp\right)\right)>EV\left(GL_{2}\left(gp\right)\right)$, $GL_{1}\left(gp\right)>GL_{2}\left(gp\right)$; (2)if $EV\left(GL_{1}\left(gp\right)\right)=EV\left(GL_{2}\left(gp\right)\right)$, if $DD\left(GL_{1}\left(gp\right)\right)=DD\left(GL_{2}\left(gp\right)\right)$, then $GL_{1}\left(gp\right)=GL_{2}\left(gp\right)$; if $DD\left(GL_{1}\left(gp\right)\right)<DD\left(GL_{2}\left(gp\right)\right)$, then, $GL_{1}\left(gp\right)>GL_{2}\left(gp\right)$.

Definition 5 [70]. If $GL=\left\{gl_{g\alpha }|g\alpha =-g\theta ,\cdots ,-2,-1,0,1,2,\cdots g\theta \right\}$ is LTSs, $GL_{1}\left(gp\right)=\left\{g{l_{1}}^{\left(g\varphi \right)}\left(g{p_{1}}^{\left(g\varphi \right)}\right)|\;g\varphi =1,2,\cdots ,\#GL_{1}\left(gp\right)\right\}$ and $GL_{2}\left(gp\right)=\left\{g{l_{2}}^{\left(g\varphi \right)}\left(g{p_{2}}^{\left(g\varphi \right)}\right)|\;g\varphi =1,2,\cdots ,\#GL_{2}\left(gp\right)\right\}$ be two PLTSs, with $\#GL_{1}\left(gp\right)=\#GL_{2}\left(gp\right)=\#GL\left(gp\right)$, then probabilistic linguistic Euclidean distance $PLED\left(GL_{1}\left(gp\right),GL_{2}\left(gp\right)\right)$ is constructed in Eq. (6):

(6) $$PLED\left(GL_{1}\left(gp\right),GL_{2}\left(gp\right)\right)=\sqrt{\frac{\sum_{g\varphi =1}^{\#GL\left(gp\right)}{\left(g{p_{1}}^{\left(g\varphi \right)}gh\left(g{l_{1}}^{\left(g\varphi \right)}\right)-g{p_{2}}^{\left(g\varphi \right)}gh\left(g{l_{2}}^{\left(g\varphi \right)}\right)\right)}^{2}}{\#GL\left(gp\right)}}$$

## 3 PL-LogTODIM-PROMETHEE technique for PL-MAGDM

### 3.1 PL-MAGDM problem description

**Fig. 1:**
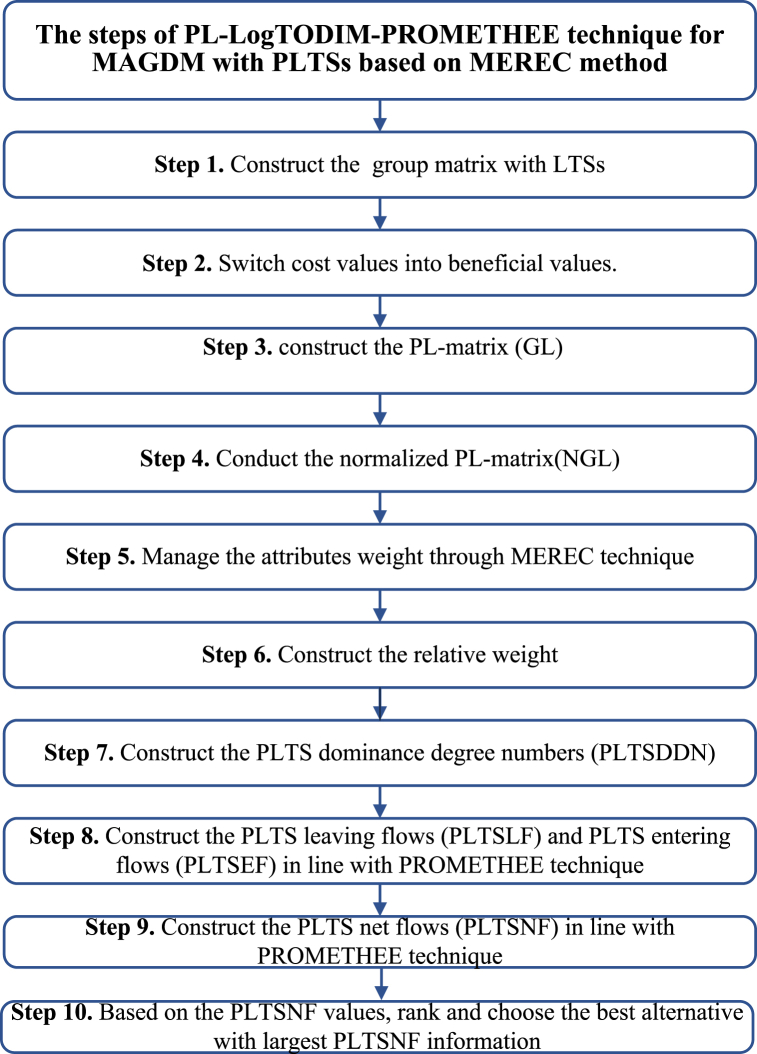
The steps of PL-LogTODIM-PROMETHEE technique for MAGDM with PLTSs based on MEREC method.

The PL-LogTODIM-PROMETHEE technique is constructed for MAGDM. Let $=\left\{GA_{1},GA_{2},\cdots ,GA_{m}\right\}$ , $GG=\left\{GG_{1},GG_{2},\cdots ,GG_{n}\right\}$ be alternatives and attributes with attribute weight gw, where $gw_{j}\in \left[0,1\right]$, $\sum_{j=1}^{n}gw_{j}=1$ and a family of experts $GE=\left\{GE_{1},GE_{2},\cdots ,GE_{q}\right\}$. Suppose that the LTSs are constructed as: $l_{ij}^{\left(gk\right)}$ . Then, PL-LogTODIM-PROMETHEE technique is constructed for PL-MAGDM, **please see** Appendix A **(**Figure 1**).**

Step 1 Construct the LTSs as: $gl_{ij}^{\left(gk\right)}$.

Step 2 Switch cost values into beneficial values. If the cost value is $gl_{\vartheta }$, then the beneficial one is $gl_{-g\vartheta }\left(g\vartheta =-3,-2,-1,0,1,2,3\right)$.

Step 3 Switch the $gl_{ij}^{\left(gk\right)}$ into PLTs: $g{l_{ij}}^{\left(g\varphi \right)}\left(g{p_{ij}}^{\left(g\varphi \right)}\right),g\varphi =1,2,\cdots ,\#GL_{ij}\left(gp\right)$ and construct the PL-matrix $GL={\left(GL_{ij}\left(gp\right)\right)}_{m\times n}$, $GL_{ij}\left(gp\right)=\left\{g{l_{ij}}^{\left(g\varphi \right)}\left(g{p_{ij}}^{\left(g\varphi \right)}\right)|\;g\varphi =1,2,\cdots ,\#GL_{ij}\left(gp\right)\right\}$.

Step 4 Conduct the normalized PL-matrix $NGL={\left(GL_{ij}\left(gp\right)\right)}_{m\times n}$, $NGL_{ij}\left(gp\right)=\left\{\begin{array}{l} g{l_{ij}}^{\left(g\varphi \right)}\left(g{p_{ij}}^{\left(g\varphi \right)}\right)\\ |\;g\varphi =1,2,\cdots ,\#NGL_{ij}\left(gp\right) \end{array}\right\}$.

### 3.2 Manage the attributes weight through MEREC technique

Step 5 Manage the attributes weight through MEREC technique.

MEREC technique [71] is a useful technique to manage the weight value.

(1) Manage the normalized probabilistic linguistic deviation degree matrix $NPLDDM_{ij}$ is constructed in Eq. (7):

(7) $$NPLDDM_{ij}=\frac{\min_{i}\left(DD\left(GL_{ij}\left(gp\right)\right)+EV\left(GL_{ij}\left(gp\right)\right)\right)}{\left(DD\left(GL_{ij}\left(gp\right)\right)+EV\left(GL_{ij}\left(gp\right)\right)\right)}$$

(2) Manage the overall performance $NPLDDM_{i}$ in Eq. (8).

(8) $$NPLDDM_{i}=\ln\left(1+\left(\frac{1}{n}\sum_{j=1}^{n}\left|\ln\;NPLDDM_{ij}\right|\right)\right)\text{.}$$

(3) Manage the performance of $GA_{i}$ through removing every attribute in Eq. (9).

(9) $$NPLDDM_{i\left(j\right)}=\ln\left(1+\left(\frac{1}{n}\sum_{k=1,k\neq j}^{n}\left|\ln\;NPLDDM_{ik}\right|\right)\right),i=1,2,\cdots ,m;k=1,2,\cdots ,n\text{,}$$

(4) Manage the summation of PLTS absolute deviations number (PLTSADN) in Eq. (10):

(10) $$PLTSADN_{j}=\sum_{i=1}^{m}\left|NPLDDM_{i\left(j\right)}-NPLDDM_{i}=\right|\text{,}$$

(5) Manage the weight values in Eq. (11):

(11) $$gw_{j}=\frac{PLTSADN_{j}}{\sum_{j=1}^{n}PLTSADN_{j}},j=1,2,\cdots n\text{.}$$

### 3.3 PL-LogTODIM-PROMETHEE technique for PL-MAGDM

The PL-LogTODIM-PROMETHEE technique is constructed for MAGDM.

Step 6 Construct the relative weight in Eq. (12):

(12) $$rgw_{j}=gw_{j}/\max_{j}gw_{j}\text{,}$$

Step 7 Construct the PLTS dominance degree numbers (PLTSDDN).

(1) The $PLTSDDN_{j}\left(GA_{i},GA_{t}\right)$ of $GA_{i}$ over $GA_{t}$ for $GG_{j}$ is constructed in line with LogTODIM technique in Eq. (13).

(13) $$PLTSDDN_{j}\left(GA_{i},GA_{t}\right)=\left\{ \begin{array}{cc} \frac{rgw_{j}\times \log\left(1+10\rho PLED\left(GL_{ij}\left(gp\right),GL_{tj}\left(gp\right)\right)\right)}{\sum_{j=1}^{n}rgw_{j}} & \text{if}\;EV\left(GL_{ij}\left(gp\right)\right)>EV\left(GL_{tj}\left(gp\right)\right)\\ 0 & \text{if}\;EV\left(GL_{ij}\left(gp\right)\right)=EV\left(GL_{tj}\left(gp\right)\right)\\ -\frac{rgw_{j}\times \lambda \;\log\left(1+10\rho PLED\left(GL_{ij}\left(gp\right),GL_{tj}\left(gp\right)\right)\right)}{\sum_{j=1}^{n}rgw_{j}} & \text{if}\;EV\left(GL_{ij}\left(gp\right)\right)<EV\left(GL_{tj}\left(gp\right)\right) \end{array}\right.$$

where λ∈[1,5] and $\rho \in N^{+}$ is done in light with agent's perception [62].

(2) The $PLTSDDN_{j}\left(GA_{i}\right)\left(j=1,2,\cdots ,n\right)$ with respect to $GG_{j}$ is constructed in Eq. (14):

(14) $$\begin{array}{l} PLTSDDN_{j}\left(GA_{i}\right)={\left[PLTSDDN_{j}\left(GA_{i},GA_{t}\right)\right]}_{m\times m}\\ \begin{array}{cccc} \;GA_{1} & \;GA_{2} & \cdots & \;GA_{m} \end{array}\\ =\begin{array}{c} GA_{1}\\ GA_{2}\\ \vdots \\ GA_{m} \end{array}\left[\begin{array}{cccc} 0 & PLTSDDN_{j}\left(GA_{1},GA_{2}\right) & \cdots & PLTSDDN_{j}\left(GA_{1},GA_{m}\right)\\ PLTSDDN_{j}\left(GA_{2},GA_{1}\right) & 0 & \cdots & PLTSDDN_{j}\left(GA_{2},GA_{m}\right)\\ \vdots & \vdots & \cdots & \vdots \\ PLTSDDN_{j}\left(GA_{m},GA_{1}\right) & PLTSDDN_{j}\left(GA_{m},GA_{2}\right) & \cdots & 0 \end{array}\right] \end{array}$$

(3) Produce the $PLTSDDN_{j}\left(GA_{i}\right)$ for $GG_{j}$ in Eq. (15):

(15) $$PLTSDDN_{j}\left(GA_{i}\right)=\sum_{t=1}^{m}PLTSDDN_{j}\left(GA_{i},GA_{t}\right)$$

(4) The $PLTSDDN={\left(PLTSDDN_{it}\right)}_{m\times m}$ is constructed in Eq. (16):

(16) $$PLTSDDN={\left(PLTSDDN_{it}\right)}_{m\times m}=\left[\begin{array}{ccccc} & GA_{1} & GA_{2} & \ldots & GA_{m}\\ GA_{1} & \sum_{j=1}^{n}PLTSDDN_{j}\left(GA_{1},GA_{1}\right) & \sum_{j=1}^{n}PLTSDDN_{j}\left(GA_{1},GA_{2}\right) & \ldots & \sum_{j=1}^{n}PLTSDDN_{j}\left(GA_{1},GA_{m}\right)\\ GA_{2} & \sum_{j=1}^{n}PLTSDDN_{j}\left(GA_{2},GA_{1}\right) & \sum_{j=1}^{n}PLTSDDN_{j}\left(GA_{2},GA_{2}\right) & \ldots & \sum_{j=1}^{n}PLTSDDN_{j}\left(GA_{2},GA_{m}\right)\\ \vdots & \vdots & \vdots & \vdots & \vdots \\ GA_{m} & \sum_{j=1}^{n}PLTSDDN_{j}\left(GA_{m},GA_{1}\right) & \sum_{j=1}^{n}PLTSDDN_{j}\left(GA_{m},GA_{2}\right) & \ldots & \sum_{j=1}^{n}PLTSDDN_{j}\left(GA_{m},GA_{m}\right) \end{array}\right]$$

Step 8 Construct the PLTS leaving flows (PLTSLF) and PLTS entering flows (PLTSEF) in line with PROMETHEE technique in Eq. (17), (18).

(17) $$PLTSLF\left(GA_{i}\right)=\frac{1}{m-1}\sum_{t=1}^{m}PLTSDDN_{it},i=1,\ldots ,m\text{.}$$

(18) $$PLTSEF\left(GA_{i}\right)=\frac{1}{m-1}\sum_{k=1}^{m}PLTSDDN_{kt},i=1,\ldots ,m\text{.}$$

Step 9 Construct the PLTS net flows (PLTSNF) in line with PROMETHEE technique in Eq. (19).

(19) $$PLTSNF\left(GA_{i}\right)=PLTSLF\left(GA_{i}\right)-PLTSEF\left(GA_{i}\right),i=1,2,\ldots ,m$$

Step 10 Based on the PLTSNF values, rank and choose the best alternative with largest PLTSNF information.

## 4 Numerical example and comparative analysis

### 4.1 Numerical example

The cooperation between schools and enterprises in developed countries has emerged due to the urgent need for technical talents in the development of market economy. The government attaches great importance to vocational education, which combines theoretical and practical knowledge of students to promote industrial development and bring about a win-win situation. It is worth affirming that European and American countries attach great importance to school enterprise cooperation in terms of ideology, because this model cultivates students who are urgently needed technical talents for industrial development and have more employment options. At the same time, their legal system for school enterprise cooperation is very comprehensive, and relevant laws are continuously improved according to the needs of industrial development and economic development. In addition, the three main entities of school enterprise cooperation - the government, enterprises, and schools - cooperate closely with each other and have clear division of labor. The general government takes the lead in introducing relevant policies to lead cooperation between enterprises and schools. Enterprises and schools achieve win-win cooperation through various cooperation models such as joint development of scientific research projects, sharing of research results, and joint training of technical talents. In terms of financial security, governments in developed countries invest heavily and allocate special funds for research and development. In the past decade, the research and development funds of developed countries have accounted for over 2.5 % of the country's GDP. The key to improving the quality of cultivating composite talents and mastering the driving force of innovation is to invest more in research and development funds and produce more. In addition to government power, as school enterprise cooperation matures, other social forces are also constantly joining in. The role of intermediary institutions in promoting should not be underestimated. Some of them bring research projects from outside the school into the campus or transform research achievements into productivity to accelerate the integration of industry and education; It can also provide venture capital to promote school enterprise cooperation and accelerate the incubation of micro and small high-tech enterprises. The development evaluation of school-enterprise cooperation from the perspective of collaborative education is a MAGDM. Therefore, the development evaluation of school-enterprise cooperation from the perspective of collaborative education is presented to demonstrate the approach developed in this essay. There are five school-enterprise cooperation schemes $GA_{i}\left(i=1,2,3,4,5\right)$ to choose in line with four attributes: ①GG_1_ is the school-enterprise cooperation achievements for multiple-navigation sensor systems.②GG_2_ is school-enterprise cooperation cost from the perspective of collaborative education; ③GG_3_ is school-enterprise cooperation economic benefits from the perspective of collaborative education; ④GG_4_ is school-enterprise cooperation social benefits from the perspective of collaborative education. The school-enterprise cooperation cost from the perspective of collaborative education (GG_2_) is cost attribute, others are beneficial one. Then, the PL-LogTODIM-PROMETHEE technique is employed to manage the development evaluation of school-enterprise cooperation from the perspective of collaborative education.

Step 1 The five school-enterprise cooperation schemes $GA_{i}\left(i=1,2,3,4,5\right)$ are assessed through employing the PLTSs:

$$\begin{array}{l} GL=\{gl_{-3}=extremely\;poor\left(GEP\right),gl_{-2}=very\;poor\left(GVP\right)\text{,}\\ gl_{-1}=poor\left(GP\right),gl_{0}=medium\left(GM\right),gl_{1}=good\left(GG\right)\text{,}\\ gl_{2}=very\;good\left(GVG\right),gl_{3}=extremely\;good\left(GEG\right)\} \end{array}$$

by five DMs, as constructed in Table 1, Table 2, Table 3, Table 4, Table 5.

Step 2 Switch GG_2_ into beneficial attributes (See Table 6, Table 7, Table 8, Table 9, Table 10).

**Table 6:** Linguistic matrix for DM_1_.

Alternatives	GG_1_	GG_2_	GG_3_	GG_4_
GA_1_	GG	GG	GP	GP
GA_2_	GVG	GVP	GM	GEP
GA_3_	GG	GP	GM	GEG
GA_4_	GP	GP	GG	GG
GA_5_	GP	GVP	GEG	GP

**Table 7:** Linguistic matrix for DM_2_.

Alternatives	GG_1_	GG_2_	GG_3_	GG_4_
GA_1_	GM	GP	GG	GG
GA_2_	GVG	GVP	GG	GP
GA_3_	GG	GVG	GP	GG
GA_4_	GVG	GG	GVG	GP
GA_5_	GM	GEG	GVG	GP

**Table 8:** Linguistic matrix for DM_3_.

Alternatives	GG_1_	GG_2_	GG_3_	GG_4_
GA_1_	GVG	GP	GG	GG
GA_2_	GG	GVG	GEG	GVG
GA_3_	GM	GM	GVP	GG
GA_4_	GG	GP	GG	GG
GA_5_	GVG	GG	GP	GG

**Table 9:** Linguistic matrix for DM_4_.

Alternatives	GG_1_	GG_2_	GG_3_	GG_4_
GA_1_	GVG	GG	GVP	GP
GA_2_	GVP	GVG	GM	GVG
GA_3_	GM	GP	GG	GEG
GA_4_	GG	GP	GG	GVG
GA_5_	GVG	GG	GG	GM

**Table 10:** Linguistic matrix for DM_5_.

Alternatives	GG_1_	GG_2_	GG_3_	GG_4_
GA_1_	GG	GP	GG	GG
GA_2_	GG	GP	GVG	GP
GA_3_	GVG	GVG	GVG	GVP
GA_4_	GG	GVP	GM	GVP
GA_5_	GG	GP	GG	GM

Step 3 Conduct the PLTSs (Table 11).

**Table 11:** The PLTSs.

Alternatives	GG_1_	GG_2_
GA_1_	$\left\{gl_{1}\left(0.2\right),gl_{2}\left(0.6\right),gl_{3}\left(0.2\right)\right\}$	$\left\{gl_{1}\left(0.8\right),gl_{2}\left(0.2\right)\right\}$
GA_2_	$\left\{gl_{-1}\left(0.2\right),gl_{2}\left(0.8\right)\right\}$	$\left\{gl_{-1}\left(0.2\right),gl_{1}\left(0.6\right),gl_{2}\left(0.2\right)\right\}$
GA_3_	$\left\{gl_{-2}\left(0.2\right),gl_{0}\left(0.6\right),gl_{1}\left(0.2\right)\right\}$	$\left\{gl_{2}\left(0.6\right),gl_{3}\left(0.4\right)\right\}$
GA_4_	$\left\{gl_{0}\left(0.8\right),gl_{1}\left(0.2\right)\right\}$	$\left\{gl_{-3}\left(0.2\right),gl_{-1}\left(0.4\right),gl_{0}\left(0.4\right)\right\}$
GA_5_	$\left\{gl_{1}\left(0.8\right),gl_{2}\left(0.2\right)\right\}$	$\left\{gl_{-2}\left(0.2\right),gl_{-1}\left(0.8\right)\right\}$

Alternatives	GG_3_	GG_4_
GA_1_	$\left\{gl_{-1}\left(0.6\right),gl_{1}\left(0.4\right)\right\}$	$\left\{gl_{0}\left(0.2\right),gl_{1}\left(0.6\right),gl_{2}\left(0.2\right)\right\}$
GA_2_	$\left\{gl_{1}\left(0.6\right),gl_{2}\left(0.4\right)\right\}$	$\left\{gl_{-2}\left(0.2\right),gl_{-1}\left(0.6\right),gl_{1}\left(0.2\right)\right\}$
GA_3_	$\left\{gl_{1}\left(0.4\right),gl_{2}\left(0.4\right),gl_{3}\left(0.2\right)\right\}$	$\left\{gl_{-1}\left(0.2\right),gl_{2}\left(0.8\right)\right\}$
GA_4_	$\left\{gl_{-2}\left(0.2\right),gl_{1}\left(0.8\right)\right\}$	$\left\{gl_{1}\left(0.8\right),gl_{3}\left(0.2\right)\right\}$
GA_5_	$\left\{gl_{-1}\left(0.2\right),gl_{1}\left(0.8\right)\right\}$	$\left\{gl_{-1}\left(0.2\right),gl_{1}\left(0.6\right),gl_{2}\left(0.2\right)\right\}$

Step 4 Conduct the normalized PL-matrix (Table 12).

**Table 12:** Normalized PL-matrix.

Alternatives	GG_1_	GG_2_
GA_1_	$\left\{gl_{1}\left(0.2\right),gl_{2}\left(0.6\right),gl_{3}\left(0.2\right)\right\}$	$\left\{gl_{1}\left(0.0\right),gl_{1}\left(0.8\right),gl_{2}\left(0.2\right)\right\}$
GA_2_	$\left\{gl_{-1}\left(0.0\right),gl_{-1}\left(0.2\right),gl_{2}\left(0.8\right)\right\}$	$\left\{gl_{-1}\left(0.2\right),gl_{1}\left(0.6\right),gl_{2}\left(0.2\right)\right\}$
GA_3_	$\left\{gl_{-2}\left(0.2\right),gl_{0}\left(0.6\right),gl_{1}\left(0.2\right)\right\}$	$\left\{gl_{2}\left(0.0\right),gl_{2}\left(0.6\right),gl_{3}\left(0.4\right)\right\}$
GA_4_	$\left\{gl_{0}\left(0.0\right),gl_{0}\left(0.8\right),gl_{1}\left(0.2\right)\right\}$	$\left\{gl_{-3}\left(0.2\right),gl_{-1}\left(0.4\right),gl_{0}\left(0.4\right)\right\}$
GA_5_	$\left\{gl_{1}\left(0.0\right),gl_{1}\left(0.8\right),gl_{2}\left(0.2\right)\right\}$	$\left\{gl_{-2}\left(0.0\right),gl_{-2}\left(0.2\right),gl_{-1}\left(0.8\right)\right\}$

Alternatives	GG_3_	GG_4_
GA_1_	$\left\{gl_{-1}\left(0.0\right),gl_{-1}\left(0.6\right),gl_{1}\left(0.4\right)\right\}$	$\left\{gl_{0}\left(0.2\right),gl_{1}\left(0.6\right),gl_{2}\left(0.2\right)\right\}$
GA_2_	$\left\{gl_{1}\left(0.0\right),gl_{1}\left(0.6\right),gl_{2}\left(0.4\right)\right\}$	$\left\{gl_{-2}\left(0.2\right),gl_{-1}\left(0.6\right),gl_{1}\left(0.2\right)\right\}$
GA_3_	$\left\{gl_{1}\left(0.4\right),gl_{2}\left(0.4\right),gl_{3}\left(0.2\right)\right\}$	$\left\{gl_{-1}\left(0.0\right),gl_{-1}\left(0.2\right),gl_{2}\left(0.8\right)\right\}$
GA_4_	$\left\{gl_{-2}\left(0.0\right),gl_{-2}\left(0.2\right),gl_{1}\left(0.8\right)\right\}$	$\left\{gl_{1}\left(0.0\right),gl_{1}\left(0.8\right),gl_{3}\left(0.2\right)\right\}$
GA_5_	$\left\{gl_{-1}\left(0.0\right),gl_{-1}\left(0.2\right),gl_{1}\left(0.8\right)\right\}$	$\left\{gl_{-1}\left(0.2\right),gl_{1}\left(0.6\right),gl_{2}\left(0.2\right)\right\}$

Step 5 Conduct the weight values:

$$\begin{array}{l} gw_{1}=0.3058,gw_{2}=0.3257\\ gw_{3}=0.1318,gw_{4}=0.2367 \end{array}\text{.}$$

Step 6 Conduct the relative weight: rgw={0.9389,1.0000,0.4047,0.7267}

Step 7 Conduct the PLTSDDN (See Table 13):

**Table 13:** The $PLTSDDN={\left(PLTSDDN_{it}\right)}_{5\times 5}$.

Alternatives	GA_1_	GA_2_	GA_3_	GA_4_	GA_5_
GA_1_	0.0000	2.0258	−1.5374	−2.3615	1.1338
GA_2_	−0.2429	0.0000	1.4887	0.1889	−2.3776
GA_3_	1.7712	−2.7047	0.0000	−2.9027	1.6445
GA_4_	0.8004	−1.1181	1.8155	0.0000	0.7670
GA_5_	−1.5603	2.8643	−0.1146	−2.6491	0.0000

Step 8 Construct the PLTSLF and PLTSEF (Table 14).

**Table 14:** The PLTSLF and PLTSEF.

	PLTSLF	PLTSEF
GA_1_	0.7685	−0.7393
GA_2_	1.0673	−0.9429
GA_3_	1.6522	−2.1916
GA_4_	−7.7244	2.2648
GA_5_	1.1676	−1.4597

Step 9 Construct the PLTSNF (Table 15).

**Table 15:** The PLTSNF and order.

	PLTSNF	Order
GA_1_	1.5078	4
GA_2_	2.0102	3
GA_3_	3.8438	1
GA_4_	−9.9891	5
GA_5_	2.6273	2

Step 10 **In** line with the PLTSNF, the order is: $GA_{3}>GA_{5}>GA_{2}>GA_{1}>GA_{4}$ and $GA_{3}$ is the optimal school-enterprise cooperation scheme.

**Table 1:** Linguistic matrix for DM_1_.

Alternatives	GG_1_	GG_2_	GG_3_	GG_4_
GA_1_	GG	GP	GP	GP
GA_2_	GVG	GVG	GM	GEP
GA_3_	GG	GG	GM	GEG
GA_4_	GP	GG	GG	GG
GA_5_	GP	GVG	GEG	GP

**Table 2:** Linguistic matrix for DM_2_.

Alternatives	GG_1_	GG_2_	GG_3_	GG_4_
GA_1_	GM	GG	GG	GG
GA_2_	GVG	GVG	GG	GP
GA_3_	GG	GVP	GP	GG
GA_4_	GVG	GP	GVG	GP
GA_5_	GM	GEP	GVG	GP

**Table 3:** Linguistic matrix for DM_3_.

Alternatives	GG_1_	GG_2_	GG_3_	GG_4_
GA_1_	GVG	GG	GG	GG
GA_2_	GG	GVP	GEG	GVG
GA_3_	GM	GM	GVP	GG
GA_4_	GG	GG	GG	GG
GA_5_	GVG	GP	GP	GG

**Table 4:** Linguistic matrix for DM_4_.

Alternatives	GG_1_	GG_2_	GG_3_	GG_4_
GA_1_	GVG	GP	GVP	GP
GA_2_	GVP	GVP	GM	GVG
GA_3_	GM	GG	GG	GEG
GA_4_	GG	GG	GG	GVG
GA_5_	GVG	GP	GG	GM

**Table 5:** Linguistic matrix for DM_5_.

Alternatives	GG_1_	GG_2_	GG_3_	GG_4_
GA_1_	GG	GG	GG	GG
GA_2_	GG	GG	GVG	GP
GA_3_	GVG	GVP	GVG	GVP
GA_4_	GG	GVG	GM	GVP
GA_5_	GG	GG	GG	GM

### 4.2 Comparative analysis

Then, the PL-LogTODIM-PROMETHEE technique is compared with PL-GRA technique [72], PL-EDAS technique [73], PL-TOPSIS technique [74], PL-CODAS technique [75], PL-WASPAS technique [76], PL-ELECTRE II technique [77] and PL-MULTIMOORA technique [78]. The comparative results are constructed in Table 16.

**Table 16:** Order of different techniques.

Technique	Order
PL-GRA technique [72]	$GA_{3}>GA_{5}>GA_{1}>GA_{2}>GA_{4}$
PL-EDAS technique [73]	$GA_{3}>GA_{5}>GA_{2}>GA_{1}>GA_{4}$
PL-TOPSIS technique [74]	$GA_{3}>GA_{5}>GA_{1}>GA_{2}>GA_{4}$
PL-CODAS technique [75]	$GA_{3}>GA_{5}>GA_{2}>GA_{1}>GA_{4}$
PL-WASPAS technique [76]	$GA_{3}>GA_{5}>GA_{2}>GA_{1}>GA_{4}$
PL-ELECTRE II technique [77]	$GA_{3}>GA_{5}>GA_{2}>GA_{1}>GA_{4}$
PL-MULTIMOORA technique [78]	$GA_{3}>GA_{5}>GA_{2}>GA_{1}>GA_{4}$
PL-LogTODIM-PROMETHEE technique	$GA_{3}>GA_{5}>GA_{2}>GA_{1}>GA_{4}$

In the light with the WS coefficients model [79,80], the WS coefficient values between the PL-GRA technique [72], PL-EDAS technique [73], PL-TOPSIS technique [74], PL-CODAS technique [75], PL-WASPAS technique [76], PL-ELECTRE II technique [77], PL-MULTIMOORA method [78] and the PL-LogTODIM-PROMETHEE technique is 0.7917, 1.0000, 0.7917, 1.0000, 1.0000, 1.0000, 1.0000, respectively. The WS coefficient values shows the order of the PL-LogTODIM-PROMETHEE technique is same to the order of PL-EDAS technique [73], PL-TOPSIS technique [74], PL-WASPAS technique [76], PL-ELECTRE II technique [77] and PL-MULTIMOORA method [78]; the WS coefficient values shows the order of the PL-LogTODIM-PROMETHEE technique is slightly different to the order of PL-GRA technique [72] and PL-CODAS technique [75]. This verifies the PL-LogTODIM-PROMETHEE technique is effective.

## 5 Conclusion

In summary, the cooperation between schools and enterprises in China still faces stable financial guarantees, specialized regulatory and policy support, effective supervision of cooperation, and the formulation of cooperation evaluation standards. In particular, the outbreak of the COVID-19 has made the world face new changes that have not been seen in a century. Therefore, in addition to emphasizing the external factors of successful cooperation between schools and enterprises, colleges and universities should also have the courage to move inward, vigorously promote the reform of disciplines and majors, make it coincide with the mirror image of social development, and create a new talent training system. Putting people first, continuously exploring and developing the potential of students, introducing extracurricular enterprises to jointly cultivate and deepen the integration of industry and education, and cultivating more high-quality composite talents that are in line with the development of the new era society. The development evaluation of school-enterprise cooperation from the perspective of collaborative education is a MAGDM. Recently, the LogTODIM and PROMETHEE technique has been employed to conduct MAGDM issues. The PLTSs are used as a tool for characterizing uncertain information during the development evaluation of school-enterprise cooperation from the perspective of collaborative education. In this paper, the PL-LogTODIM-PROMETHEE technique is constructed to put forward the MAGDM under PLTSs. Finally, a numerical example for development evaluation of school-enterprise cooperation from the perspective of collaborative education is employed to conduct the PL-LogTODIM-PROMETHEE technique.

There may be some possible limitations of development evaluation of school-enterprise cooperation from the perspective of collaborative education, which could be further studied in future research: (1) It is a worthwhile research topic to apply prospect theory for development evaluation of school-enterprise cooperation from the perspective of collaborative education under PLTSs environment [[81], [82], [83]]; (2) It is also worthwhile to apply regret theory for development evaluation of school-enterprise cooperation from the perspective of collaborative education under PLTSs environment [[84], [85], [86]]; (3) Thoroughly studying the development evaluation of school-enterprise cooperation from the perspective of collaborative education, finding the connections between data, and fully utilizing the various information provided by school-enterprise cooperation can make existing evaluation schemes more comprehensive.

## Ethics declaration statement

The authors state that this is their original work, and it is neither submitted nor under consideration in any other journal simultaneously.

## Data availability

The data used to support the findings of this study are included within the article.

## Funding information

This study did not receive any funding in any form.

## CRediT authorship contribution statement

**Yuedan Hu:** Writing – original draft. **Chalermchai Panyadee:** Methodology, Data curation, Conceptualization.

## Declaration of competing interest

The authors declare that they have no known competing financial interests or personal relationships that could have appeared to influence the work reported in this paper.
